# Cryoglobulinemia: the “cold” problem in cardiac surgery, a single-center experience and a literature review

**DOI:** 10.1186/s44158-024-00141-x

**Published:** 2024-01-25

**Authors:** Pasquale Raimondo, Gianmarco Intini, Gianfranco Lauletta, Valentina Teora, Sergio Domenico Lenoci, Giovanni Rubino, Maria Arcangela Villani, Agnese Armenise, Antonia Stripoli, Giuseppe Colantuono, Nicola Di Bari, Giuseppe Fiore, Gianluca Paternoster, Salvatore Grasso

**Affiliations:** 1 Anesthesia and Intensive Care II, AOUC Policlinico Di Bari, Bari, Italy; 2 Division of Anesthesia and Intensive Care, University of Bari, Bari, Italy; 3 Department of Precision and Regenerative Medicine and Ionian Area, University of Bari, Bari, Italy; 4 Division of Cardiac Surgery, AOUC Policlinico Di Bari, Bari, Italy; 5 Cardiovascular Anesthesia and Intensive Care, San Carlo Hospital Potenza, Potenza, Italy

**Keywords:** Cardiac anesthesia, Cardiac surgery, Cryoglobulinemia, Extracorporeal perfusion, Intraoperative complication

## Abstract

Cardiac surgery with cardiopulmonary bypass (CBP) is essential for different cardiac procedures in order to perform surgery with a clear sight field.

To safely perform surgery with CPB and preserve brain, kidney, and patient tissue from ischemic damage, cold cardioplegia, and mild to deep hypothermia are induced during the operation.

Cryoglobulinemia is a hematological/infective-related disease (in certain cases idiopathic) in which temperature-dependent antibodies tend to aggregate and form emboli in the vascular system causing tissue damage if exposed to low temperature.

The patient with cryoglobulinemia (known and unknown) can be at risk of a major ischemic event during CPB and induced hypothermia.

This article’s aim is to evaluate the present scientific literature in order to understand how, in years, the therapeutic or preventive approach, is evolving, and to analyze and make improvements to the management of a cryoglobulinemic patient who must undergo elective or emergency cardiac surgery.

In the last part of our article, we expose our single-center experience during a 32-month-long period of survey.

In all cases, our medical team (anesthesiologists, perfusionists, and cardiac surgeons) opted for a normothermic cardiopulmonary bypass to lower the risk of cryoglobulin-associated complications.

In our experience, along with therapeutic intervention to lower the cryoglobulin titer, normothermic management of cardiopulmonary bypass is as safe as hypothermic management.

Notwithstanding our results, further studies with a larger population are needed to confirm this perioperative management in a cardiac surgery setting.

## Introduction

### Cryoglobulinemia

In 1933, Wintrobe and Buell [1] first described the phenomenon of protein precipitation at low temperatures in a serum sample from a patient with multiple myeloma. These proteins reversibly precipitate in vitro below 37 °C and redissolve at body temperature and were defined “cryoglobulins” by Lerner and Watson in 1947 [2]. Cryoglobulins are immunoglobulins that, according to their immunochemical composition, are classified as single-type cryoglobulins (SC) and mixed-type cryoglobulins (MC). Classification details are in Table 1.

**Table 1:** Cryoglobulin classification [3, 4]

	Type	Characteristic
Single type cryoglobulins **(SC)**	Type I	Monoclonal IgM, IgG, rarely IgA
Mixed type cryoglobulins **(MC)**	Type II	Monoclonal IgM(k) with rheumatoid factor activity
	Type III	Policlonal IgM–IgG immunocomplexes

Small amounts of serum cryoglobulins, asymptomatic and devoid of clinical consequences, are occasionally detected in several infectious, hematologic, and immunologic diseases, and even in a few normal serums. The term cryoglobulinemic vasculitis refers to a well-defined illness whose clinical and pathological features are pathogenetically related to the occurrence of serum cryoglobulins. In consideration of the wide spectrum of cryoglobulinemia, diagnostic and classification criteria have been proposed in order to better define this clinical condition [5, 6].

The disease prevailed in women with an F/M ratio of 2.13 [7]. For many years MC remained of undefined etiology and therefore was called “essential”. In 1990, Pascual et al. reported the observation that many cryoglobulinemic sera were anti-HCV positive [8]. Several reports in the following years confirmed this striking association [9–12]. Cryoglobulinemic vasculitis (CV) can be defined as a chronic, immune complexes-mediated systemic vasculitis, involving small and less frequently medium-sized vessels, representing the most typical extrahepatic manifestation of chronic HCV infection. According to the Revised International Chapel Hill Consensus Conference Nomenclature of Vasculitides, CV belongs to the subgroup “*immune complex small vessel vasculitis*” together with IgA vasculitis and hypocomplementemic urticarial vasculitis [13]. If anti-HCV antibodies and HCV RNA can be detected in > 90% of cryoglobulinemic patients, cryoglobulins are detectable in about 25% of HCV chronically infected patients; why only 10–15% of them develop a clear CV is still debated, probably reflecting genetic and/or environmental factors [14].

Less commonly, MC has been found to be associated with HCV-negative connective tissue diseases or other viral or bacterial infections including HIV, HBV, and HEV [14]. Therefore, the term “essential” should be referred to a few patients in whom the etiology remained persistently undefined [15].

MC is considered a rare disease (i.e., prevalence in the general population < 5/10,000) even if some studies showed a slightly higher prevalence [16, 17].

Cryoglobulins can be diagnosed by storing a serum sample at 4 °C; if present, usually within 48–72 h (maximum 1 week), cryoglobulins will appear as a whitish precipitate at the bottom of a Wintrobe’s tube, and their amounts are quantified as the percentage of the whole serum. This value is called cryocrit, by analogy of hematocrit; its quantification is commonly related to clinical severity and can be used to assess the response to treatment [18].

CV is a systemic vasculitis whose clinical picture is characterized by a wide spectrum of symptoms ranging from the sporadic appearance of cutaneous purpura to severe life-threatening conditions. The triad “*purpura/arthralgia/weakness*” occurs in the majority of cases and is considered a hallmark of CV. Cutaneous manifestations can vary from palpable purpura of lower limbs to chronic torpid ulcers usually located at the supramalleolar regions. Other skin reactions are represented by Raynaud’s phenomenon, livedo reticularis, urticaria, and edema. Arthralgias usually involve the hands, knees, and ankles symmetrically. Kidney injury may complicate CV in almost 30% of cases. An indolent course with hypertension, proteinuria, microhematuria, and red blood cell casts occur in about 50% of cases, whereas nephritic or nephrotic syndromes are less common (14% and 21% of cases, respectively) and a defined picture of cryoglobulinemic glomerulonephritis evolving into chronic renal failure may involve about 14% of cases. Nervous system involvement in the course of CV ranges from 16 to 60%. Peripheral nervous system damage is usually represented by sensory-motor neuropathy of the lower limbs, paresthesia, loss of strength, pain, and burning sensations. Central nervous system alterations are less common, usually characterized by transient dysarthria, hemiplegia, and confusional state. As expected, liver damage characterized by chronic hepatitis with or without cirrhosis occurs in almost 70% of cases of HCV-related CV. Less common clinical pictures of CV are represented by gastrointestinal (2–6%) and pulmonary (5%) involvement. Intestinal ischemia or perforation should be suspected in the case of acute abdominal pain; symptoms that mimic cholecystitis and/or pancreatitis have been also described. Interstitial pneumopathy can be diagnosed in CV patients displaying dyspnea and dry cough; pictures of acute alveolar hemorrhage with hemoptysis, respiratory failure, and multiple pulmonary infiltrates are rare [14–19].

CV-induced heart failure, reversible dilated cardiomyopathy, and hypertrophic cardiomyopathy have been also reported probably due to myocardial vessel involvement. The clinical features of hyperviscosity syndrome can be recognized in 10–15% of patients with type I cryoglobulinemia but they are rare in those with type II and even rarer in those with type III MC. Symptoms are usually characterized by blurred vision, recurrent epistaxis, headache, tinnitus, vertigo, and dizziness. In severe forms, ataxia, confusion, and heart failure may be present. Funduscopic examination may reveal tortuosity or “sausage-like” appearance of the involved vessels, marked retinal venous engorgement, and retinal hemorrhage [14].

The main therapeutic goals in CV are represented by the eradication of HCV infection, deletion of the underlying B cells’ clonal expansions, and depletion of cryoproteins. For many years, clinically active CV was treated with immunosuppressive/cytotoxic drugs and steroids in different ways due to the heterogeneity and variable severity of the disease, with few or partial and transient clinical benefits. After the identification of HCV as the main etiologic factor, antiviral therapy has become of paramount importance. Interferon or pegylated interferon-based therapy with and without ribavirin has represented for many years the gold standard for the therapy of HCV-associated CV, resembling the standard of care for chronic hepatitis. A dramatic positive therapeutic impact was reached after the introduction of new direct antiviral agents against HCV with virological responses in nearly 100% of cases and complete clinical and immunological responses of CV in 40–95% of cases according to different reports [20]. Rituximab, a monoclonal antibody recognizing CD20 molecules expressed on B-cells surface, has been extensively employed for B cell depletion in patients resistant to or relapsing after conventional antiviral therapy. Its use is currently indicated in patients with persistent cryoglobulinemia despite sustained virological response [14].

Life-threatening conditions are represented by acute renal failure with oligo-anuria, rapidly progressive renal failure, diffuse alveolar hemorrhages, acute cerebrovascular and cardiovascular events, ischemic colitis, sepsis complicating skin ulcer infection, and critical liver failure.

In these cases, Therapeutic Plasma Exchange (TPE) plays a crucial role as emergency treatment in that large amounts of immune complexes can be rapidly removed from the bloodstream, representing a treatment of choice [21], followed by rituximab administration and/or high-dose (or pulsed) glucocorticoids. Patients should receive direct-acting agents (DAAs) treatment as soon as possible when their condition is compatible with antiviral therapy completion [22]. Double-filtration plasmapheresis has been also employed in the treatment of CV patients with inveterate, indolent leg ulcers, achieving progressive scarring until complete wound healing after subsequent procedures [23]. Therapeutic plasma exchange is also useful in type I cryoglobulinemia associated with hematological diseases like multiple myeloma, particularly in the case of severe vasculitis and hyperviscosity syndrome [24].

## Cryoglobulinemia and cardiac surgery: the cold problem

Since the success of open-heart surgery with extracorporeal circulation introduced by Gibbons in 1953, cardiopulmonary bypass (CBP) technology has become a fundamental part of cardiovascular surgery procedures [25].

Extracorporeal circulation with the CPB system implies a great variety of effects on an organism, and the knowledge required for optimal management is quite extensive [26].

Among these effects, hypothermia to prevent brain, heart, and tissue damage is important, but it has some consequences at different levels, such as hemostatic and hemorrhagic problems. For these reasons, international guidelines have been developed for optimal temperature management during CPB [27].

Hypothermic cardiac surgery may be a situation at risk for cryoglobulins vasculitis in patients with HCV, immune, and/or hematological disease. Cryoprecipitation during or after cardiac surgery with hypothermic cardiopulmonary bypass is susceptible to starting vessel inflammation and major ischemic events, such as skin ulcerations, neuropathies, glomerulonephritis, or arthritis, among the most frequently associated co-morbidities [28].

We conducted a literature review on PubMed, Google Scholar, Cochrane Library database from 2000 to October 2023, using the Boolean operator, searching for the following keywords: “*Cryoglobulinemia*” AND “*Cardiac Surgery*” and, after ruling out irrelevant and out-of-topic articles, we included all the articles describing the management of cardiopulmonary bypass in patients with known and unknown cryoglobulin disease.

We excluded all the articles published before 2000 because the manuscripts were incomplete or not in English.

The aim of this brief review is to emphasize the lack of protocol for the management of patients with cryoglobulinemia in cardiac surgery. In 2013, in a brief review of the electronic records of the Mayo Clinic, Barbara et al. [29] proposed a perioperative approach tailored to thermal amplitude and cryoglobulins titers in a series of 16 patients (from 2003 to 2010) affected by Cold Hemagglutinin Disease who underwent cardiac procedures requiring cardiopulmonary bypass.

Since then, there has been very little literature about these topics:

### Valve surgery and ascending aorta replacement

Regarding valve replacement and aortic dissection/aneurism, the surgery that most of all requires careful temperature management, we have found four different case reports with different comorbidities treated with different levels of hypothermia during cardiopulmonary bypass.

Fontana and colleagues [28] proposed a tailored approach in a patient with HIV–HCV-related cryoglobulin who underwent a replacement of the ascending aorta with valvuloplasty according to Tirone David technique. They assessed the temperature-dependent differential cryoprecipitation of serum (the titer of Cryoglobulin at different temperatures) and found the titer to decrease above 20 °C, so, during CPB, they maintained the core temperature of the patient around 22–24 °C, using a mild hypothermia to minimize the risk of cryoprecipitation, an acceptable compromise according to their operative protocol.

As a second option, Yamazaki and colleagues, instead, performed a successful deep hypothermia in patients with Stanford type A aortic dissection which required cardiac arrest and deep hypothermia for neuroprotection [30]. In this case, a 64-year-old man diagnosed with symptomatic idiopathic cryoglobulinemia underwent immunosuppressive therapy plus plasma exchange and double filtration plasmapheresis; with the abovementioned therapies the qualitative analysis of cryoglobulins was found negative, hence, when the patient experienced chest and back pain and was diagnosed with aortic dissection, the medical team opted for deep hypothermia (minimal nasopharyngeal temperature was 17.3 °C) with selective cerebral perfusion. These authors concluded that with appropriate therapy and a negative qualitative cryoglobulin test, cardiopulmonary bypass under hypothermia can be safely considered.

Satomi and colleagues [31], using a third different approach, provide an example of successfully performed aortic valve replacement with normothermic CBP in an 82-year-old woman with aortic stenosis, Waldeström’s Macroglobulinemia and hyperviscosity syndrome, positive for qualitative test for cryoglobulins; preoperative blood testing demonstrated that the patient’s blood tended to gel below 32 °C. In this case, a forced air warming system was used to maintain the rectal temperature between 36 and 37 °C during extracorporeal circulation, moreover, blood temperature in the arterial and venous cannulas was kept between 36 to 37.5 °C and 35 to 36 °C respectively. Cardiac arrest was induced with normothermic cardioplegia and maintained with continuous perfusion of warm blood cardioplegia via the coronary sinus at 30 to 70 ml/min (temperature was always kept between 35 and 36.5 °C). In a similar way, Edmiston and colleagues [32] in a recent paper published in October 2023, performed a mitral valve surgery on a 61-year-old patient with type II cryoglobulinemia and endocarditis; they successfully performed a normothermic systemic perfusion with intermittent cold blood cardioplegia after two rounds of preoperative plasma exchange session.

At last, as described by Vela and colleagues [33], a patient with cryoglobulinemia undergoing cardiac surgery for combined mitral and aortic endocarditis, had several complications despite clinical and pharmacological interventions. The patient had remote hepatitis C (HCV) infection with an undetectable viral load and HCV-RNA. Blood cultures revealed Streptococcus viridians and transesophageal echocardiography showed a perforated aortic valve cusp and mitral valve vegetation with associated regurgitation. Preoperatively he underwent two rounds of plasma exchange and apheresis. Warm cardioplegia was used in the operating theatre but, in the postoperative period, he developed a fever nonresponding to conventional treatment. Physical cooling was applied and purpura fulminans with heart failure occurred. The patient underwent VA ECMO as rescue therapy, but he was elected for withdrawal of life-sustaining measures.

### Coronary artery bypass graft

Regarding Chronic or Acute coronary disease three cases were reported by Faikh et al. [34], Saifullah et al. [35], and Panagiotoupulos et al. [36].

Faikh et al. [34] discuss a case of a 59-year-old patient with an established diagnosis of idiopathic cryoglobulinemia treated with plasmapheresis, rituximab, and oral corticosteroids. He had a history of multiple leukocytoclastic vasculitic skin lesions, gangrene of the right first and second toe, and left index finger requiring amputations. The patient underwent coronary artery bypass grafting for a three-vessel disease; at admission, a qualitative test for cryoglobulins was positive, and for this, he underwent two sessions of plasmapheresis. During surgery patient was under CPB using 31 °C blood cardioplegia and mild hypothermic temperature (minimum nasopharyngeal and bladder temperature during the procedure was 32.4 °C). No cryoglobulin-related complication was reported.

Saifullah and colleagues [35], more recently, described a similar case of a 74-year-old man with idiopathic Cryoglobulinemia who required Coronary Artery Bypass Graft (CABG) and mitral valve repair; these authors emphasize a multidisciplinary setting and performed (according with other specialists) perioperative tests with plasmapheresis and steroids therapy both pre- and post-operatively, using a warm cardioplegia and normothermic extracorporeal circulation intraoperatively.

In December 2022, Panagiotoupulos et al. [36] published a recent case report in literature with a CABG of a 57-year-old man with no story of Cryoglobulinemia or Cold hemagglutinin disease. CPB was performed as usual when, suddenly, blood clots appeared on the surgical field. The patient was promptly warmed up with warm saline infusion and progressive normothermia and extubated ten hours after the procedure with no cold agglutinin/cryoglobulin-related complications.

In addendum, as an anecdotic case, Antonacci et al. [37] report a case of sudden pulmonary embolism in a 63‐year‐old male with a history of cryoglobulinemia and Raynaud’s phenomenon undergoing surgery for the removal of a poorly differentiated thymic carcinoma. The patient underwent emergent surgical embolectomy with midline sternotomy access and CPB. Right and left pulmonary arteries were incised, removing a significant number of fresh emboli.

The patient was easily weaned from CPB and discharged 11 days post-procedure.

Revised literature is listed in Table 2.

**Table 2:** Literature review

Year	Authors	Procedure	Scheduled or Urgent	Comorbidity	Pre-operative testing	Temp	Cardioplegia	Normothermia	Other treatment	Outcome
2001	Takeishi et al	Atrial myxoma	---	Proteinuria, Raynaud phenomenon Purpura	None	Hypothermic	Cold	No	None	Positive
2006	Fontana et al	Tirone David	Scheduled	HIV/HCV	None	22–24 °C	Cold	No	None	Positive
2017	Fakih et al	Coronary artery bypass graft	Scheduled	Idiopathic cryoglobulinemia chronic kidney disease, hypertension, coronary artery disease	Cryo +	32.4 °C	Warm	Yes	Plasmapheresis pre-op, Steroids pre-op, Immunosuppressive therapy pre-op	Positive
2017	Satomi et al	Aortic valve replacement	Scheduled	Hypertension, Waldestrom’s Macroglobulinemia	Cryo +	37 °C	Warm	Yes	None	Positive
2019	Vela et al	Mitral + aortic valve replacement	Urgent	HCV, endocarditis	Cryo +	37 °C	Warm	Yes	Plasma Exchange/Plasmapheresis pre-op, Steroids post-op	Negative
2020	Yamazaki et al	Ascending aorta replacement	Urgent	Idiopathic cryoglobulinemia	None	17 °C	Cold	No	Plasma Exchange/Plasmapheresis pre-op, Steroids pre-op, Immunosuppressive therapy pre-op	Positive
2020	Saifullah et al	Mitral valve repair + coronary artery bypass graft	Scheduled	Idiopathic cryoglobulinemia, diabetes, hypertension, hypercholesterolemia	Cryo +	34 °C	Warm	Yes	Plasmapheresis pre and post-op, Steroids pre-op	Positive
2022	Panagiotopoulos et al	Coronary artery bypass graft	Scheduled	Diabetes, hypercholesterolemia	None	Hypothermic	From Cold to Warm	No	None	Positive
2023	Edmiston et al	Mitral valve Substitution	Scheduled	Type II cryoglobulinemia, cryoglobulinemic glomerulonephritis (renal failure stage III)	Cryo +	Normothermic	Cold	Yes	Plasma exchange pre-op, Chronic Steroids pre-op, Immunosuppressive therapy pre-op	Positive

## Discussion

In this brief literature review, we have found case reports with different management and outcomes. Most of the case reports describe patients with symptomatic and clear diagnoses of cryoglobulinemia who were scheduled for elective surgery. Many patients had hematologic malignancies or HCV/HIV infection and, in three cases, were diagnosed with preoperatory idiopathic cryoglobulinemia.

The outcome was positive in patients who were early diagnosed with cryoglobulinemia or had suggestive symptoms; in a single case reported by Vela et al. a patient with known cryoglobulinemia, died of late complications not directly connected with the procedure. As described in the anecdotic case report by Antonacci et al. the only patient with unknown cryoglobulinemia had major intraoperative complications.

The suggested approaches we found in the literature are composed of preoperative and intraoperative practices and are listed in Table 3.

**Table 3:** Perioperative management for cryobulinemic patients

Cardiopulmonary bypass and cardioplegia management strategies for cryoglobulinemia
**Preoperative management**
-Plasmapheresis and/or plasma exchange [30, 32–35]
-Immunosuppressive therapy [30, 32, 34]
-Steroids [30, 32–35]
**Intraoperative management**
-Warm/cold blood cardioplegia and systemic warming [31–33, 35]
-Warm**/**cold blood cardioplegia and mild hypothermia **(temperature above cryoprecipitation threshold)** [28, 34]
-Hypothermia after negative cryo test [30]

### Preoperative management

The approach was in five cases (all with known cryoglobulinemia) conducted with a preoperatory plasmapheresis to lower the qualitative and quantitative titer of cryoglobulins, in five cases, patients were or had been under steroid treatment prior to or after the operation. In three cases, the patient received immunosuppressive therapy.

### Intraoperative management

The intraoperative management differs between different case reports; Yamazaki and colleagues performed a deep hypothermic cardiopulmonary bypass after two rounds of plasmapheresis with a negative qualitative cryoglobulin test and Fontana et al. conducted the extracorporeal circulation with mild hypothermia after having established the temperature–dependent cryoprecipitation, maintaining the patient temperature during cardiopulmonary bypass in a “safe zone”. In another case report the procedure was conducted using a normothermic CPB; authors applied a forced warming to the patient during the CPB using active warming, warming of the arterial and venous cannulas, and either continuous or intermittent warm cardioplegia.

### Our center experience

This study was conducted in our regional specialized hospital for cardiac surgery, Azienda Ospedaliera–Universitaria Consorziale Policlinico di Bari (Italy), for 32 months (2020–2022). Our inclusion criteria were known or unknown diagnosis of cryoglobulinemia in patients admitted for on-pump cardiac surgery.

#### Study population

We enrolled only four patients, one man, and three women. All patients who meet the inclusion criteria had known cryoglobulinemia. Three patients were admitted with a clear diagnosis; one patient was diagnosed during our routine preoperative assessment. The mean age of patients in our case series was 72.5 years, while the median 71.5 years. At admission one of four patients had autoimmune disease as a comorbidity, three had hematologic diseases, and none of the patients were positive for HCV.

No complications were recorded during the CPBs. The mean temperature of the CPBs was 36.75 °C. The mean post-surgery ICU stay was 9.75 days, while the median 7 days. The outcome was favorable for all enrolled patients.

Laboratory data are reported in Table 4.

**Table 4:** Study population: laboratory data

	Patient	WBC (× 10^3^/uL)	CRP (mg/L)	PLT (× 10^3^/uL)	LACT (mmol/L)	Creatine (mg/dL)	CRYOC (%)
Pre-operative data	1	4.9	33.4	126	1.4	0.91	Presence (< 0.5)
	2	6.3	1	278	1.5	1.5	Presence (< 0.5)
	3	14.24	115	254	2	3.41	Presence (< 0.5)
	4	7.04	8.9	298	0.9	6.47	Presence (< 0.5)
Post-operative data	1	10.05	75.6	130	1.6	1.22	Presence (< 0.5)
	2	8.96	41.8	147	3	1.33	Presence (< 0.5)
	3	18.35	81.3	152	4.5	3.16	Presence (< 0.5)
	4	15.51	94.7	257	1.2	4.94	Presence (< 0.5)
Discharge data	1	5.46	8	279	1	1.04	Presence (< 0.5)
	2	4.6	4.4	356	1	1.73	Presence (< 0.5)
	3	8.66	45.1	196	1.2	1.63	Presence (< 0.5)
	4	9.93	76.6	394	0.9	6.23	Presence (< 0.5)

The only clinical sign of cryoglobulinemia manifested in our case series was preoperative renal damage in terms of chronic kidney failure (stages II/III/IV, according to KDIGO classification) in three of the four patients.

In our center, there is no specific technical protocol for the treatment of patients with known pathology, but each case treated was discussed and decided upon by the whole cardiac surgical team considering the hemodynamic condition of the patient and his comorbidities. The average temperature used during CPB was 36.75 °C, which denotes a preference for implementing the normothermic technique.

## Conclusion

In our review and clinical experience, we have found that treatment options for patients undergoing cardiopulmonary bypass are approximately the same approaches suggested since 1991: double filtration plasmapheresis, (DFPP) pre- and postoperatively as well as during extracorporeal circulation [38], normothermic cardiopulmonary bypass with continuous warm blood cardioplegia [39], or preoperative plasmapheresis and steroid therapy [40, 41].

Concerning the risks of a normothermic CPB, a systematic literature review by Kwok M. Ho et al. [42] found that the use of normothermic CPB is as safe as hypothermic surgery and is associated with a lower risk of allogenic blood transfusion. This makes normothermic CPB a promising and, above all, safe solution for the management of patients with cryoglobulinemia.

Further studies are needed to assess safer and evidence-based management of patients with cryoglobulinemia needing cardiac surgery.

## Data Availability

Yes.

## References

[1] Wintrobe MM, Buell MV (1933). Hyperproteinemia associated with multiple myeloma. Bull Johns Hopkins Hosp.

[2] Lerner AB, Watson CJ (1947). Studies of cryoglobulins. I. Unusual purpura associated with the presence of a high concentration of cryoglobulin (cold precipitable serum globulin). Am J Med Sci..

[3] Brouet JC, Clauvel JP, Danon F, Klein M, Seligmann M (1974). Biologic and clinical significance of cryoglobulins. Areport of 86 cases. Am J Med..

[4] Abel G, Zhang QX, Agnello V (1993). Hepatitis C virus infection in type II mixed cryoglobulinemia. Arthritis Rheum.

[5] Quartuccio L, Isola M, Corazza L, Ramos-Casals M, Retamozo S, Ragab GM, Zoheir MN (2014). Validation of the classification criteria for cryoglobulinemic vasculitis. Rheumathology.

[6] Damoiseaux J (2014). The diagnosis and classification of the cryoglobulinemic syndrome. Autoimm Rev.

[7] Lauletta G, Russi S, Conteduca V, Sansonno L, Dammacco F, Sansonno D (2013). Impact of cryoglobulinemic syndrome on the outcome of hepatitis C virus infection: a 15 year prospective study. Medicine (Baltimore).

[8] Pascual M, Perrin L, Giostra E, Schifferli JA (1990). Hepatitis C virus in patients with cryoglobulinemia type II. J Infect Dis.

[9] Ferri C, Greco F, Longombardo G, Palla P, Moretti A, Marzo E (1991). Antibodies to hepatitis C virus in patients with mixed cryoglobulinemia. Arthritis Rheum.

[10] Agnello V, Chung RT, Kaplan LM (1992). A role for hepatitis C virus infection in type II cryoglobulinemia. N Engl J Med.

[11] Dammacco F, Sansonno D, Cornacchiulo V, Mennuni C, Carbone R, Lauletta G (1993). Hepatitis C virus infection and mixed cryoglobulinemia: a striking association. Int J Clin Lab Res.

[12] Cacoub P, Fabiani FL, Musset L, Perrin M, Frangeul L, Leger JM (1994). Mixed cryoglobulinemia and hepatitis C virus. Am J Med.

[13] Jennette JC, Falk RJ, Bacon PA (2013). 2012 revised International Chapel Hill Consensus Conference Nomenclature of Vasculitides. Arthritis Rheum.

[14] Dammacco F, Lauletta G, Russi S, Leone P, Tucci M, Manno C, Monaco S, Ferrari S, Vacca A, Racanelli V (2019). Clinical practice: hepatitis C virus infection, cryoglobulinemia and cryoglobulinemic vasculitis. Clin Exp Med.

[15] Galli M, Oreni L, Saccardo F (2017). HCV-unrelated cryoglobulinaemic vasculitis: the results of a prospective observational study by the Italian Group for the Study of Cryoglobulinemias (GISC). Clin Exp Rheumathol.

[16] Monti G, Saccardo F, Castelnovo L, Novati P, Sollima S, Riva A (2014). Prevalence of mixed cryoglobulinemia syndrome and circulating cryoglobulins in a population-based survey: the Origgio study. Autoimmun Rev.

[17] (2017) Meeting of the International Task Force for Disease Eradication, June 2017. Wkly Epidemiol Rec 92(37):537–56. English, French. PMID: 28914042. https://pubmed.ncbi.nlm.nih.gov/28914042/28914042

[18] Lauletta G, Russi S, Conteduca V, Sansonno L (2012). Hepatitis C virus infection and mixed cryoglobulinemia. Clin Dev Immunol.

[19] Ramos-Casals M, Stone JH, Cid MC, Bosch X (2012). The cryoglobulinemias. Lancet.

[20] Mazzaro C, Dal Maso L, Mauro E, Visentini M, Tonizzo M, Gattei V, Andreone P, Pozzato G (2020). Hepatitis C virus-related cryoglobulinemic vasculitis: a review of the role of the new direct anriviral agents (DAAs) therapy. Autoimmun Rev.

[21] Marson P, Monti G, Montani F, Riva A, Mascia MT, Castelnovo L, Filippini D, Capuzzo E, Moretto M, D’Alessandri G, Marenchino D, Zani R, Fraticelli P, Ferri C, Quartuccio L, De Silvestro G, Oreni L, Accorsi P, Galli M (2018). Apheresis treatment of cryoglobulinemic vasculitis: a multicentre cohort study of 159 patients. Transfus Apher Sci..

[22] Galli M, Monti G, Marson P, Scaini P, Pietrogrande M, Candela M, Castelnovo L, Faggioli P, Novati P, Zani R, Mascia MT, Saccardo F, Mazzaro C, Sarzi-Puttini P, Sebastiani M, Quartuccio L, De Vita S (2019). Recommendations for managing the manifestations of severe and life-threatening mixed cryoglobulinemia syndrome. Autoimmun Rev.

[23] Ramunni A, Lauletta G, Brescia P, Saliani MT, Montrone M, Chironna M, Sansonno D, Dammacco F, Coratelli P (2008). Double-filtration plasmapheresis in the treatment of leg-ulcers in cryoglobulinemia. J Clin Apher.

[24] Solimando AG, Sportelli A, Troiano T, Demarinis L, Di Serio F, Ostuni A, Dammacco F, Vacca A, Ria R (2018) A multiple myeloma that progressed as type I cryoglobulinemia with skin ulcers and foot necrosis. a case report. Medicine (Baltimore) 97. 10.1097/MD.0000000000012355 10.1097/MD.0000000000012355 PMC6181604 30278513

[25] Nosé Y, Motomura T, Kawahito S (2001). The ICMT publication on artificial organs volume II: Oxygenator -Artificial Lung- Past, Present, and Future.

[26] Gravlee GP, Davis RF, Stammers AH, Ungerleider RM (2008). Cardiopulmonary bypass: principles and practice.

[27] Engelman R, Baker RA, Likosky DS, Grigore A, Dickinson TA, Shore-Lesserson L, Hammon JW (2015) The society of thoracic surgeons, the society of cardiovascular anesthesiologists, and the American Society of ExtraCorporeal Technology: clinical practice guidelines for cardiopulmonary bypasstemperature management during cardiopulmonary bypass JECT. J ExtraCorporeal Technol 47:145–154 PMC4631211 26543248

[28] Fontana MD, Patrick Ruchat MD, Judith Horisberger MD, Vincent Aubert MD, Carole Mayor MD, Francois Spertini MD (2006). Prevention of cryoprecipitation during cardiopulmonary bypass in a patient with HIV-HCV co-infections. Perfusion.

[29] Barbara David W, Mauermann William J, Neal James R, Abel Martin D, Schaff Hartzell V, Winters Jeffrey L (2013). Cold agglutinins in patients undergoing cardiac surgery requiring cardiopulmonary bypass J Thorac Cardio- vasc Surg.

[30] Yamazaki K, Minatoya K, Sakamoto K, Kitagori K, Okuda M, Murakami K (2020). Hypothermic circulatory arrest for aortic dissection with cryoglobulinemia. J Card Surg..

[31] Satomi S, Kasai A, Hamaguchi E, Tsutsumi YM, Tanaka K (2017) Normothermic Cardiopulmonary Bypass in Patient With Waldenström's Macroglobulinemia and Cryoglobulinemia: A Case Report. A A Case Rep 9(6):162–163. 10.1213/XAA.0000000000000555 10.1213/XAA.0000000000000555 28520564

[32] Edmiston M, Darehzereshki A, Badhwar V, Hunter Mehaffey J (2023). Morgantown, WVa Perioperative management of cryoglobulinemia in patients requiring cardiac surgery. JTCVS Techniques..

[33] Vela RJ, Krueger A, Huffman LC, Bajona P (2019) Cryoglobulinemia: caution for patients undergoing cardiac surgery. Asian Cardiovasc Thorac Ann 27(7):600–602. 10.1177/0218492319862162. 10.1177/0218492319862162 31284726

[34] Fakih HAM, MD, Emmanuel Elueze MD, PhD, Rajiv Vij MD,  (2016). Coronary artery bypass grafting in a patient with active idiopathic cryoglobulinemia: revisiting the issue. Journal of Community Hospital Internal Medicine Perspectives.

[35] Saifullah Mohamed MD, Akshay J (2020). Patel MD, Yassir Iqbal MD, Khurum Mazhar MD and Timothy R. Graham MD Operative consideration in patient with cryoglobulinaemia undergoing cardiac surgery with use of cardiopulmonary bypass Journal of Surgical Case Reports.

[36] Ioannis Panagiotopoulos MD, Francesk Mulita MD, Georgios-Ioannis Verras MD, Anastasia Katinioti MD, Angelos Samaras MD, Konstantinos Tasios MD, Konstantinos Bouchagier MD, KonstantinosTriantafyllou MD (2022). Cold Reactive Proteins in Cardiovascular Surgery Mater Sociomed.

[37] Antonacci F, Masiglat LJT, Borrelli E, Salati M, D'Armini AM, Pelenghi S, Totaro P (2020) Acute massive pulmonary embolism during patient repositioning following excision of a thymic carcinoma in a patient affected by cryoglobulinemia. J Card Surg 35(8):2050–2052. 10.1111/jocs.14759. 10.1111/jocs.14759 32652608

[38] Kotsuka MD, Nakajima MD, Miyairi MD, Nakahara MD, Suzuki MD, Kanda MD, Irabu MD, Saito MD, Yoshida MD, Mizuno MD (1991). Coronary artery bypass grafting in a patient with cryoglobulinemia. J Cardiovasc Surg (Torino).

[39] Murata MD, Tebayashi MD, Shinozaki MD, Shimizu MD, Ito MD, Konnai MD (1995). al Successful cardiac surgery using normothermic cardiopulmonary bypass in an elderly patient with cryoglobulinemia. Nihon Kyobu Geka Gakkai Zasshi.

[40] Osada MD, Kawachi MD, Uchino MD, Hirayama MD, Ishimaru MD, Furukawa MD (1992). A successful case of thoracic aortic aneurysm with mixed cryoglobulinemia. Nihon Kyobu Geka Gakkai Zasshi.

[41] Yatsu MD, Kimura MD, Muraoka MD, Tsuboo MD, Ishihara MD, Matsuki MD (1998). Perioperative management of a patient with cryoglobulinemia for open heart surgery. Masui.

[42] Ho KM, Tan JA (2011). Benefits and risks of maintaining normothermia during cardiopulmonary bypass in adult cardiac surgery: a systematic review. Cardiovasc Ther.

